# Disrupted resting-state functional connectivity and network topology in mild traumatic brain injury: an arterial spin labelling study

**DOI:** 10.1093/braincomms/fcad254

**Published:** 2023-09-30

**Authors:** Fengfang Li, Liyan Lu, Hui Li, Yin Liu, Huiyou Chen, Fang Yuan, Hailong Jiang, Xindao Yin, Yu-Chen Chen

**Affiliations:** Department of Radiology, Nanjing First Hospital, Nanjing Medical University, Nanjing 210006, China; Department of Radiology, Nanjing First Hospital, Nanjing Medical University, Nanjing 210006, China; Department of Radiology, Nanjing Drum Tower Hospital, The Affiliated Hospital of Nanjing University Medical School, Nanjing 210008, China; Department of Radiology, Nanjing First Hospital, Nanjing Medical University, Nanjing 210006, China; Department of Radiology, Nanjing First Hospital, Nanjing Medical University, Nanjing 210006, China; Department of Neurosurgery, Shanghai Sixth People’s Hospital Affiliated to Shanghai Jiao Tong University School of Medicine, Shanghai 200235, China; Department of Radiology, Nanjing First Hospital, Nanjing Medical University, Nanjing 210006, China; Department of Radiology, Nanjing First Hospital, Nanjing Medical University, Nanjing 210006, China; Department of Radiology, Nanjing First Hospital, Nanjing Medical University, Nanjing 210006, China

**Keywords:** mild traumatic brain injury, arterial spin labelling, functional connectivity, graph theory, module

## Abstract

Mild traumatic brain injury can cause different degrees of cognitive impairment and abnormal brain structure and functional connectivity, but there is still a lack of research on the functional connectivity and topological organization of cerebral blood flow fluctuations. This study explored the cerebral blood flow, functional connectivity and topological organization of the cerebral blood flow network in acute mild traumatic brain injury patients. In total, 48 mild traumatic brain injury patients and 46 well-matched healthy controls underwent resting-state arterial spin labelling perfusion MRI and neuropsychological assessments. The functional connectivity and topological organization of the cerebral blood flow network were analysed. Then, the correlation between the changes in cerebral blood flow network characteristics and cognitive function was explored. Acute mild traumatic brain injury patients showed decreased cerebral blood flow in the right insula and increased cerebral blood flow in the right inferior temporal gyrus and left superior temporal gyrus. Abnormal cerebral blood flow network connection patterns mainly occur in sensorimotor network, default mode network, cingulo-opercular network and occipital network-related regions. Furthermore, mild traumatic brain injury disrupted the topological organization of the whole brain, which manifested as (i) reduced global efficiency; (ii) abnormal degree centrality, betweenness centrality, nodal clustering coefficient and nodal efficiency; and (iii) decreased intermodular connectivity between the occipital network and sensorimotor network. Finally, the change in network topology was correlated with the cognitive score of the mild traumatic brain injury. This study provided evidence of abnormal functional connectivity and network topology based on cerebral blood flow in acute mild traumatic brain injury patients, revealing their potential use as early markers for mild traumatic brain injury, which may contribute to both disease diagnosis and assessment.

## Graphical Abstract

**Graphical Abstract:**
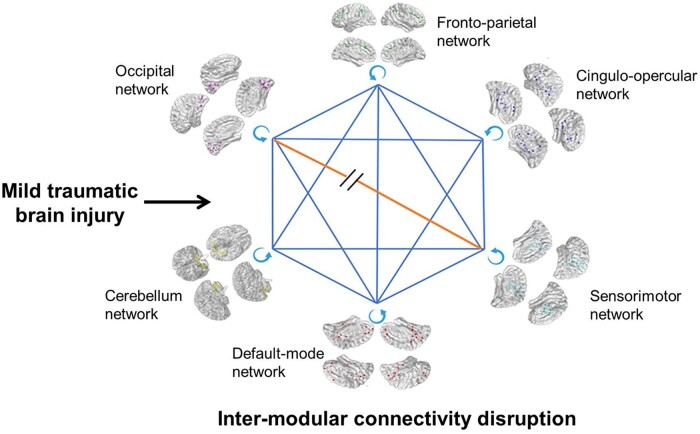

## Introduction

Mild traumatic brain injury (mTBI) is one of the most common neurological disorders and represents more than 80% of total TBI cases.^1^ mTBI may cause significant or persistent neurobehavioural and neurocognitive dysfunction, including problems with attention, memory, balance and more, and may be one of the risk factors for early dementia and psychiatric consequences.^2^ Cerebrovascular changes found after mTBI include impaired automatic regulation of the brain, namely, the ability to maintain constant cerebral blood flow (CBF) in the face of changes in blood pressure and hypotension, which may affect prognosis.^3^ Although the research and understanding of the acute and potentially long-term neurocognitive impairment of mTBI has increased, the underlying neuropathological mechanisms remain to be further elucidated.^4^ Neuroimaging studies may provide new insights into the neural mechanisms of mTBI and post-mTBI cognitive impairment.

Previous studies have shown that mTBI may induce abnormal changes in CBF autoregulation function in cerebral circulation, and cerebrovascular changes seem to contribute to the development and slow repair of secondary brain injuries.^4^ Arterial spin labelling (ASL) is a safe perfusion MRI technique that can noninvasively quantify CBF using magnetically labelled arterial blood as an endogenous contrast agent. Several studies have extensively investigated brain function and perfusion in relation to neuronal activity levels in mTBI patients and changes in resting-state CBF and CBF covariant connectivity in mTBI patients.^5^ Although recent studies have confirmed resting abnormal CBF in mTBI, these findings are somewhat varied, and no consensus has been reached. Notably, most studies using ASL showed an overall decrease in CBF in subacute and chronic mTBI patients, especially in the bilateral temporal and thalami, insula, putamen, hippocampus and left occipital and bilateral frontal cortices.^6-8^ In contrast, other findings revealed increased CBF, particularly in the temporal and frontal lobes, left striatum, left insula and dorsal anterior cingulate cortex (ACC), in mTBI.^9,10^ Additionally, various studies have shown a correlation between CBF changes and cognitive decline in mTBI patients.^11,12^ Another study reported that differential changes in CBF were associated with persistent post-concussion symptoms after mTBI.^13^

Given the well-known phenomenon that CBF is directly coupled to neuronal activity and that it fluctuates synchronously in specific brain regions that form functional networks, CBF-based studies of resting-state brain networks and functional connectivity (FC) patterns are possible^14^ and have been used to evaluate clinical populations, such as epilepsy, migraine and schizophrenia.^15-17^ However, despite the many studies that have been carried out to date, there is a lack of literature on one aspect: the study of FC and topological organization of resting CBF fluctuations in mTBI patients. Li *et al.*^10^ used the brain regions with changed CBF in acute mTBI patients as seeds, and their seed-based CBF-FC analysis found abnormal CBF covariant FC between the right superior frontal gyrus (SFG) and right parahippocampal gyrus and the left SFG and left middle occipital gyrus. Another study found sex differences in CBF covariant connectivity in certain cortical and subcortical regions in patients with acute mTBI.^18^ Therefore, the resting-state CBF and CBF-based FC changes in patients with mTBI remain unclear and need to be further evaluated. In addition, the topological characteristics of the brain network based on CBF haemodynamic variable fluctuations in mTBI patients have not been characterized by graph theory.

In the present study, based on resting-state ASL, we set out to study the changes in cerebral perfusion and FC of CBF network in acute mTBI patients, and to our knowledge, we explore for the first time the topological properties (global and local) of the CBF networks used individual CBF temporal fluctuations in the acute mTBI. In addition, we investigated the relationship between CBF connectivity and topological changes and cognitive function scores. We hypothesized that differences in CBF, CBF FC and topological organization would be observed in the acute mTBI group. We also expected that the difference might be related to cognitive function in the mTBI group.

## Materials and methods

### Participants

This study was approved by the local ethics committee of Nanjing Medical University, and all participants signed a written informed consent form prior to inclusion. MTBI was defined based on the American Congress of Rehabilitation Medicine. Forty-eight acute mTBI patients (right-handed) and 46 healthy control (HC) subjects (right-handed) participated in the current study, and the inclusion criteria of mTBI were as follows: age ≥ 20 years; initial Glasgow Coma Score (GCS) of 13–15, post-traumatic amnesia < 24 h, or loss of consciousness < 30 min. Exclusion criteria: (i) history of previous head injury, (ii) neurological or psychiatric disease, (iii) history of illicit drug or alcohol abuse, (iv) history of vascular disease or hyperthyroidism and (v) MRI contraindications. The Montreal Cognitive Assessment (MoCA) was used to quantify the clinical neurocognitive status of all participants and mainly assesses eight cognitive domains, namely, language, visuospatial/executive, attention, memory, abstraction, naming and orientation. Cognitive functional status and MRI scans with the same parameters were performed on all participants within 0–7 days after mTBI (median value = 3).

### Data acquisition

Scans were performed on a 3.0 T MRI scanner (Ingenia, Philips Medical Systems, Netherlands) using an eight-channel standard head coil. During the entire scan, headphones and pads were used to reduce scanner noise and subject movement, and subjects were asked to close their eyes, stay awake, stay still and not think about anything. ASL data were obtained using a 2D-pseudocontinuous ASL (pCASL) sequence: echo time (TE) = 11 ms; repetition time (TR) = 4000 ms; field of view (FOV) = 240 mm × 240 mm; flip angle (FA) = 90°; label duration = 1650 ms; post-label delay = 1600 ms; matrix = 64 × 64; slice thickness = 5 mm with 10% gap, and 30 control/label pairs; the ASL scan lasted for 4min and 08 s. Anatomical reference images were obtained using a 3D turbo fast-echo (3D-TFE) T1WI sequence: TE = 3.7 ms; TR = 8.1 mm; FA = 8°; thickness = 1 mm; slices = 170; gap = 0 mm; acquisition matrix = 256 × 256; and FOV = 256 mm × 256 mm, total scan duration = 5min and 28 s. Susceptibility weighted imaging (SWI) used 3D gradient echo (GRE) sequence: TE = 34 ms; TR = 22 mm; slice thickness = 1 mm; FA = 20; matrix = 276 × 319; and FOV = 220 mm × 220 mm. The fluid attenuated inversion recovery (FLAIR) used inverse recovery (IR) sequence: TR/TE = 7000/120 ms; FOV = 230 mm × 230 mm; acquisition matrix = 356 × 151; section thickness = 6 mm; slices = 18; FA = 90°; and intersection gap = 1.3 mm. SWI and FLAIR were used to investigate the presence of traumatic lesions. Patients with visible traumatic lesions were excluded.

### Image processing and CBF quantification

The pre-processing was performed with Statistical Parametric Mapping (SPM8, http://www.fil.ion.ucl.ac.uk/spm). For ASL images, the perfusion CBF maps were obtained using the ASL data processing toolbox ASLtbx (https://cfn.upenn.edu/). The detailed procedures have been described in a previous study.^19^ Briefly, the pre-processor follows these steps: motion correction (translations and rotations < 2 mm and 2°), co-registration with structural images, temporal filtering, normalization to Montreal Neurological Institute (MNI) standard space (resolution: 2 × 2 × 2 mm^3^), and full-width at half-maximum (FWHM) Gaussian kernel for spatial smoothing (FWHM = 6 mm). To evaluate regional CBF differences between groups, the CBF data were normalized (=voxel CBF/global grey matter CBF) to obtain normalized CBF maps. In addition, to correct for the effects of partial or atrophy volume effects, the grey matter volume (GMV) of the voxel was statistically analysed as a co-variable. GMV was calculated using SPM8, and the detailed process has been described in previous studies.^20^

### Resting-state FC of the CBF network

In the SPM8 (http://www.fil.ion.ucl.ac.uk/spm) and GRETNA toolbox [Version 2.0.0, http://www.nitrc.org/projects/gretna/ (accessed on 30 October 2020)], a pre-processing process mainly includes skull dissection, normalization, slice time correction and motion correction. Linear regression with a bandpass filter (0.008–0.09 Hz) was used for denoising to remove the mean signal in white matter and CSF.^21^ Then, for FC analysis, Pearson correlation analysis was performed by selecting the average filtering time series of anatomical and functional ROIs from the DOS-160 template and the GRETNA toolbox, respectively. They used a total of 160 ROIs (160 × 160 network with 12 720 edges) as seeds. To test for significant differences in FC between groups, correlation coefficients were further transformed into *z*-values by using Fisher’s *r* to *z* transformation.

### Network metrics

The Gretna toolbox (Version 2.0.0, http://www.nitrc.org/projects/gretna) based on MATLAB R2013b was used for network metrics analysis. In our study, the brain network is considered to be binarized in the sense that all edges are assumed to represent relationships of equal strength between nodes and undirected in the sense that it summarizes symmetric relationships between nodes. Based on previous studies,^22^ to exclude spurious and weak connections, the threshold range of sparsity was determined to be 0.03–0.50 in increments of 0.01, and only positive relationships were considered. To avoid specific threshold selection, the area under the curve (AUC) method, which has been widely used in graph theory-based network research, was adopted.

All network metrics used in the current study include global and nodal metrics. The details are as follows: global network metrics include global efficiency (*Eg*) and local efficiency (*Eloc*); nodal metrics include characteristic path length (*Lp*), clustering coefficient (*Cp*), degree centrality (*DC*), node efficiency, betweenness centrality (*BC*) and modularity. Details on the use and interpretation of these network metrics have been reported in previous literature.^23^ In brief, *Eg* is a measure of the global transmission capacity of a network. *Eloc* is a measure of the fault tolerance ability of the network, which represents the information exchange ability of each subgraph after removing the index node. *Cp* reflects the efficiency of the network in exchanging information at the cluster level and is a measure of the number of connections between a node and its nearest node (neighbour). *Lp* is the average of all shortest path lengths between all node pairs and measures the degree of overall routing efficiency. The *DC* refers to a measure of the connectivity between different nodes connected to a node. The *BC* is a measure that depends on constructing graphs into path lengths.

For modular analysis, the 160 ROIs based on the Dos-160 template were divided into six subnetworks as six modules, which included the default mode network (DMN), fronto-parietal network (FPN), sensorimotor network (SMN), cingulo-opercular network (CON), occipital network (ON) and cerebellum network (CN). Module assignment provides the basis for evaluating patterns of intermodule and intramodule connectivity at the module level. The intramodule connections and intermodule connections at the module level are calculated as follows:

Intramodule connectivity (*C_s_*) is defined as follows:


$$C_{s}=\;\frac{\sum_{i,j\in_{s}}\;\varepsilon_{ij}}{N_{s}\times (N_{S\;}-1)}.$$

Here, *N_s_* represents the number of nodes in module *s*, and ***ε****_i,j_* represents the existing edges in module *s*.

The intermodule connectivity (*C_s,t_*) between modules *s* and *t* is defined as follows:


$$C_{s,t}=\frac{\sum_{i\in_{s,j}\in_{t}}\;\varepsilon_{i,j}}{N_{s}\times N_{t}},$$

where *N_t_* refers to the number of nodes in module *t*, *N_s_* represents the number of nodes in module *s*, and *E_i_*,*_j_* is the existing edge between modules *t* and *s*.

### Statistical analysis

Group differences in demographic data were analysed by independent sample *t*-test (mean) and chi-square test (proportion), and *P* < 0.05 was considered to be significant. Group differences in CBF, FC and network metrics were analysed by two-sample *t*-test and corrected for multiple comparisons using the false discovery rate (FDR) (*P* < 0.05 was considered to be significant). We also performed Spearman’s correlation analyses between altered network properties and cognitive performance, controlling for sex, age, education years and individual mean GMV. FDR correction was also used to correct for multiple comparisons. SPSS Statistics 20.0 (IBM Corporation, Armonk, NY, USA) and the GRETNA toolbox were used for statistical analysis (*P* < 0.05 was considered to be significant).

## Results

### Clinical and demographic characterization

As shown in Table 1, in this study, all 48 acute mTBI patients and 46 HCs were included. There was a good demographic match between groups, such as gender (*P* = 0.666), age (*P* = 0.212) and education level (*P* = 0.663). The MoCA scores of the mTBI groups were significantly lower than those of the HC group (*P* < 0.001). For cognitive variables, the patients with acute mTBI showed lower visuospatial/executive (*P* = 0.001) and memory (*P* = 0.002) scores. However, no significant differences were found in the scores of other cognitive variables between groups (*P* > 0.050).

**Table 1:** Demographic characteristics and cognitive performance in patients with mTBI and HCs

Characteristic	mTBI (*n* = 48)	Controls (*n* = 46)	*P*-value
Age (years)	42.25 ± 10.31	39.57 ± 10.30	0.212
Education(years)	12.25 ± 3.08	12.91 ± 3.19	0.663
Sex (female/male)	25/23	26/20	0.666
MoCA scores	24.60 ± 1.94	26.46 ± 1.62	0.000*
Visuospatial/executive	3.73 ± 0.86	4.28 ± 0.68	0.001*
Naming	2.83 ± 0.37	2.89 ± 0.31	0.421
Attention	5.65 ± 0.69	5.78 ± 0.46	0.270
Language	2.46 ± 0.58	2.61 ± 0.57	0.212
Abstraction	1.75 ± 0.60	1.89 ± 0.37	0.178
Memory	2.44 ± 1.16	3.24 ± 1.19	0.002*
Orientation	5.75 ± 0.43	5.76 ± 0.43	0.904

Data are the mean ± standard deviation; *P < 0.05.

### Group differences in resting-state quantitative CBF

**Figure 1:**
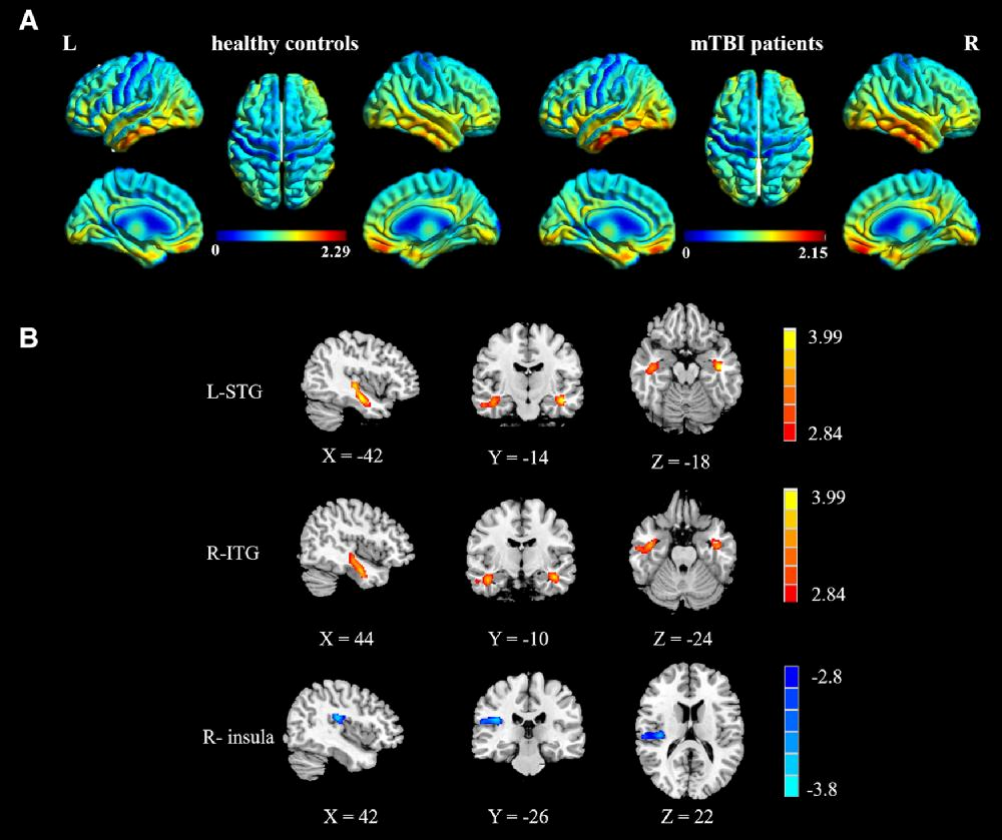
**CBF features**. (**A**) Quantitative maps of CBF for mTBI patients and HCs. (**B**) Brain regions showing significant differences in normalized CBF between mTBI patients and HCs. R, right; L, left.

Figure 1A shows the CBF maps of the HC group and mTBI group. Figure 1B and Table 2 show the normalized CBF differences between groups at the voxel-based level. Compared with HCs, patients with acute mTBI showed increased normalized CBF in the left superior temporal gyrus (STG) and right inferior temporal gyrus (ITG). In contrast, the patients with acute mTBI also had decreased CBF in the right insula.

**Table 2:** Brain regions with significant group differences in normalized CBF

Brain regions	BA	Peak MNI coordinates *x*, *y*, and *z* (mm)	Peak T value	Cluster size (voxels)
**mTBI > HC**				
R_ITG	20	44, −10, −24	3.8045	500
L_ STG	20	−42, −14, −18	4.2187	475
**mTBI < HC**				
R_insula	-	42, −26, 22	−3.8596	373

Abbreviations: R, right; L, left.

### CBF FC patterns and network topological metrics

**Figure 2:**
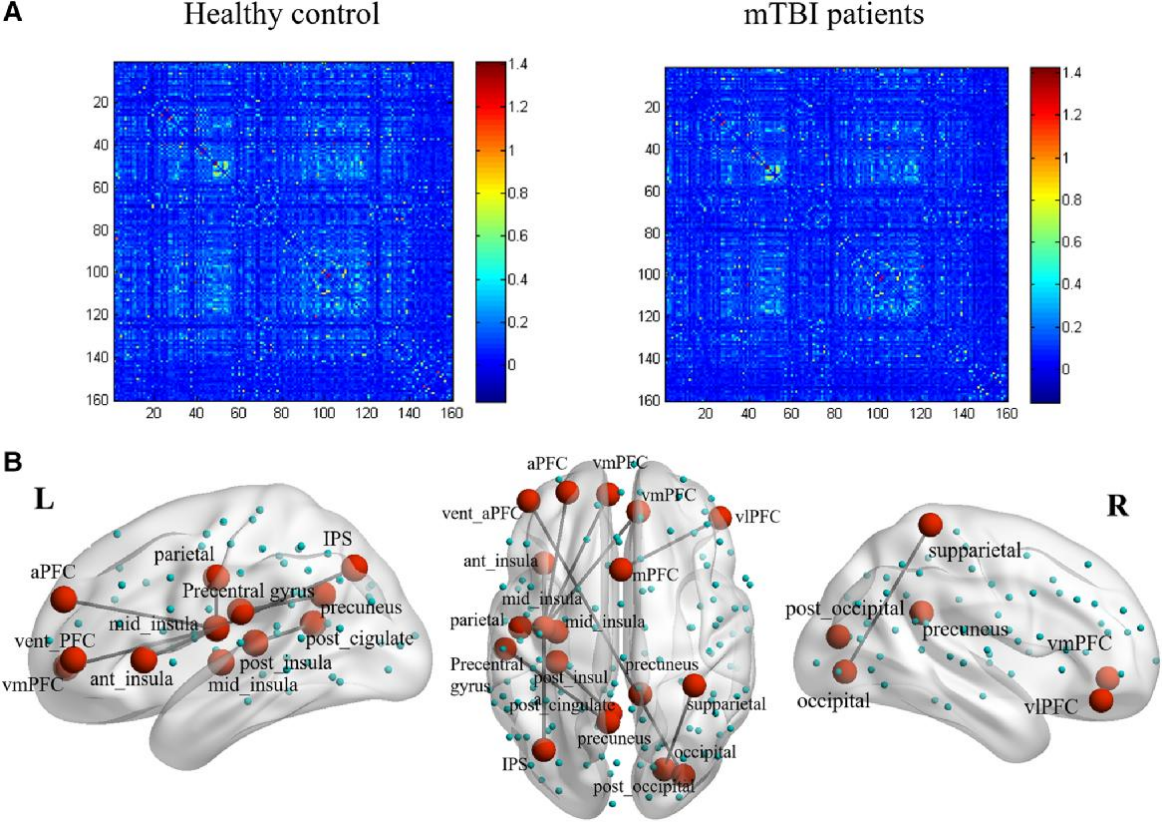
**CBF functional connectivity patterns**. (**A**) Mean functional connectivity matrices of mTBI patients and HCs group, involving 160 nodes interconnected by 12 720 edges. (**B**) Whole-brain CBF-network connectivity differences between groups. R, right; L, left. IPS, intraparietal sulcus; vmPFC, ventromedial prefrontal cortex; vlPFC, ventrolateral prefrontal cortex; aPFC, anterior prefrontal cortex.

The average connection matrix involving all 160 anatomic nodes (joined by 12 720 edges) revealed 10 significantly altered functional connections (involved the DMN-, SMN-, ON- and CON-related regions) (*P* < 0.05, FDR corrected) that significantly differed between mTBI patients and HCs, as exemplified in Fig. 2. Specifically, acute mTBI patients had increased FC between the left insula and bilateral prefrontal cortex (PFC) and parietal lobe regions and the left post-cingulate between the PFC and occipital regions. In addition, mTBI patients also had increased FC between the left precuneus and left precentral gyrus and between the right superior parietal and right occipital regions.

**Figure 3:**
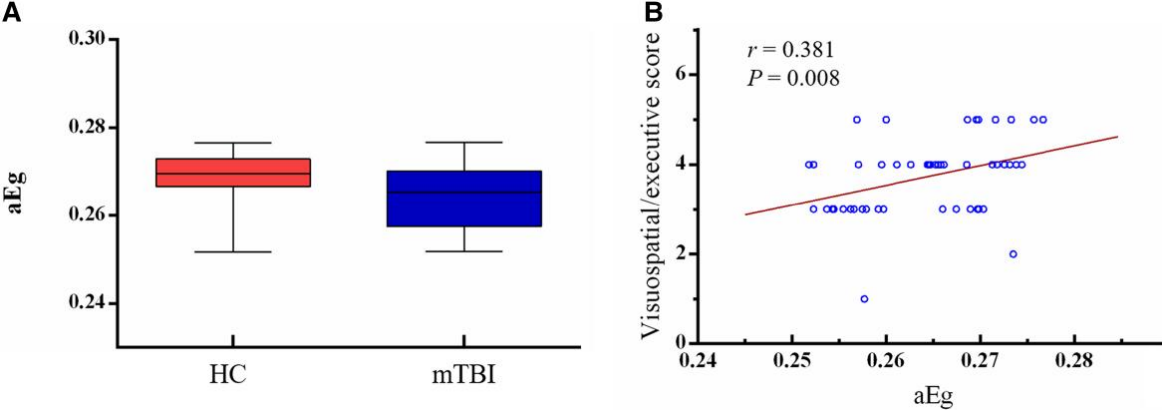
**Global proprieties and correlations**. (**A**) Significant difference in global efficiency between mTBI and HCs group (two sample *t*-test, *t* = 2.221, *P* = 0.029). (**B**) Significant positive correlation between the global efficiency and visuospatial/executive score. The error bar represents the min to max. *aEg*, global efficiency.

**Figure 4:**
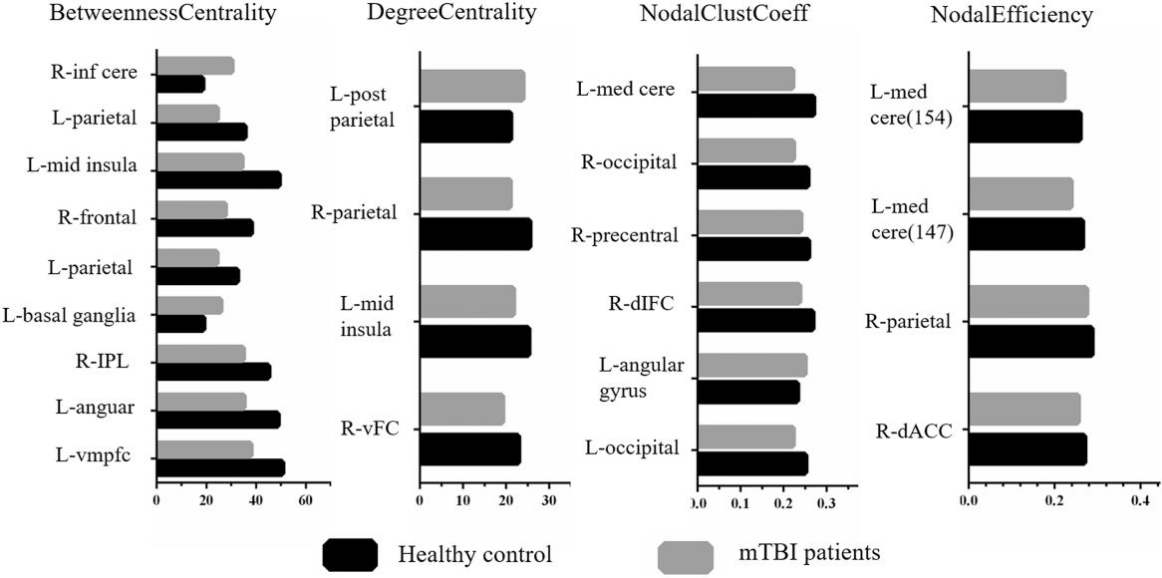
**Nodal proprieties**. Brain regions with significant differences in nodal properties (betweenness centrality, degree centrality, nodal efficiency and nodal cluster coefficient) between mTBI patients and HCs (two-sample *t*-test, *P* < 0.05, FDR corrected). L, left; R, right. The bar and error bar represent the mean values and 95%CI of the network properties. IPL, inferior parietal lobule; vmpfc, ventromedial prefrontal cortex; vFC, ventral frontal cortex; dIFC, dorsal inferior frontal cortex; dACC, dorsal anterior cingulate cortex.

For global proprieties, quantitative between-group comparisons showed that only *Eg* exhibited a significant decrease compared with HC (*t* = 2.221, *P* = 0.029) (Fig. 3A). For nodal proprieties, significant differences in BC, DC, nodal efficiency and nodal *Cp* were found between groups (Fig. 4).

### CBF functional intramodule and intermodule integration

**Figure 5:**
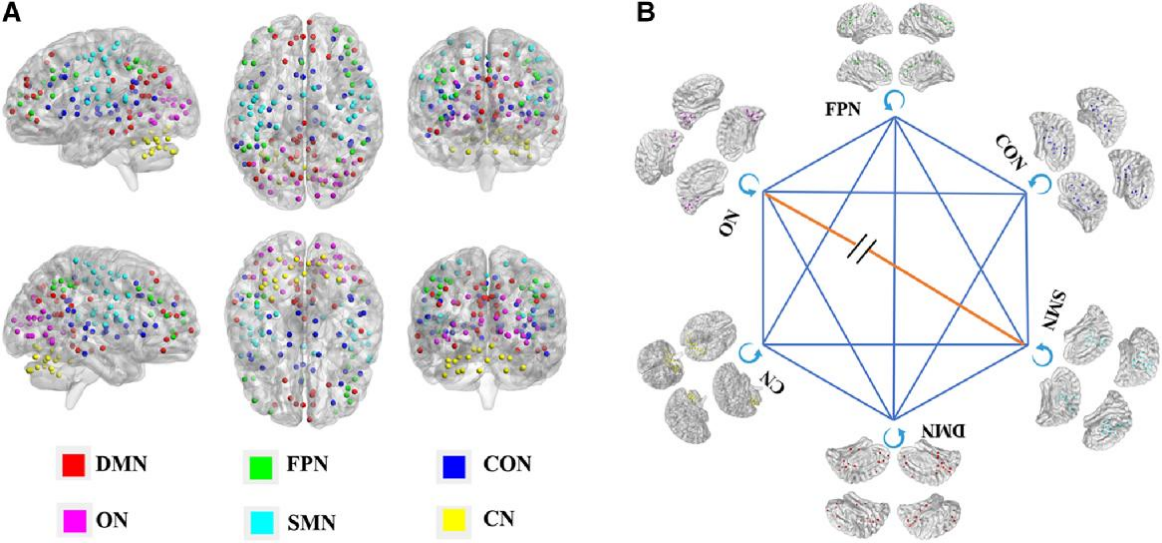
**The effects of mTBI on intermediate modular structure**. Six modules were identified based on Dos-160 template (**A**) including the DMN, SMN, FPN, CON, ON and CN. (**B**) The intraconnectivity and interconnectivity for all six *a priori* modules.

Figure 5A showed the six modules based on the Dos-160 template. Figure 5B shows the intraconnectivity and interconnectivity for all six *a priori* modules. Based on this modular architecture, we compared intramodular connections in six brain networks between groups, and no significant differences were observed in the intramodular connectivity of the six brain networks between the two groups (all *P* > 0.05). We also detected a difference in intermodular connectivity between groups, and we observed significantly reduced intermodular connectivity between the ON and SMN (*t* = −2.443, *P* = 0.016) in the acute mTBI group compared with HCs.

### CBF network relationships to cognitive performance

For the quantitative CBF and network topological metrics, focusing on the regions and metrics that showed between-group differences, we only observed a significant positive correlation between *Eg* and the visuospatial/executive score (*r* = 0.381, *P* = 0.008) in the mTBI group (Fig. 3B). We also correlated the module-level network metrics of six brain networks of interest (i.e. DMN, SMN, CON, FPN, ON and CN) and cognitive performance scores. There was no other significant correlation in mTBI and HC group (*P* > 0.05). Moreover, there were no surviving correlations after FDR correction (*P* > 0.05).

## Discussion

This study reported a CBF-based functional neuroimaging analysis examining cerebral haemodynamic and FC changes in acute mTBI patients using resting-state ASL data and evaluated the topological organization of the CBF network. Compared with HCs, acute mTBI patients demonstrated abnormal CBF in the insula and temporal regions. For the FC of the CBF network, prominent changes were localized in the PFC, insula and occipital lobes. In addition, significant differences in *Eg* and some nodal proprieties matrix were observed between groups. In particular, our results showed that the intermodular connectivity between the ON and SMN was significantly reduced in the acute mTBI group.

Abnormalities in the insula and prefrontal lobes have been widely reported in mTBI.^4^ The insula, also known as the limbic integration cortex, plays a key role in integrating internal and external processes, emotional processing, cognitive control and behaviour.^24,25^ Although previous studies have found changes in insula structural and FC during different disease courses of mTBI,^26^ these findings are still inconsistent and provide little information on the pathophysiology of early disease. In contrast, studies of acute mTBI have certain advantages in identifying the changes in brain structure and function during the onset, thus providing important information on the pathogenesis of mTBI. Abnormal changes in CBF in the insula are consistent with previous studies based on functional MRI (fMRI),^27,28^ suggesting that this may be one of the core regions for the onset of acute mTBI. In addition, acute mTBI also had abnormal CBF in frontal regions, especially the medial prefrontal gyrus, and increased CBF in the bilateral temporal lobes. Stephens *et al*.^9^ found that CBF of the left insula was increased in adolescent athletes after 2–6 weeks of mTBI compared with controls. Doshi *et al*.^29^ found increased CBF in the occipital and frontal lobes within 10 days after mTBI. However, the neuropathological mechanism of increased CBF remains unclear, and it has been suggested that the increased local CBF in mTBI patients may represent a neuroprotective response to meet the metabolic demands of brain tissue during injury and repair.^30^ Taken together, these results suggest that the insula, prefrontal and temporal lobes may play key neuropathological roles in the pathogenesis of acute mTBI. Our results based on ASL provide an important contribution to this evidence, as our study included data from acute mTBI patients who had never received specific treatment after mTBI and thus can provide us with key information on early changes after mTBI.

The human brain is a highly optimized network connection system that is conducive to distributed and specialized processing and can be quantified by certain graph theoretic metrics.^14^ Using these measures, the present study firstly found the existence of large-scale CBF connectivity patterns based on ASL and demonstrated changes in network properties in mTBI. Although changes in the *Eg* of functional brain networks have been reported in previous fMRI studies.^31^ Here, we found that the *Eg* of the CBF network in acute mTBI was reduced, which further verified the disruption of global information transmission in acute mTBI patients. The result may also reflect disruptions in neuronal integration between distributed regions, as shown by disruptions in intermodular connections, which play an important role in orchestrating neural activity throughout the brain. In addition, the correlation between *Eg* and cognitive performance after mTBI suggests that EG has neurocognitive significance in recognizing and predicting mTBI-related network behaviours. For the nodal characteristics, the brain regions with significant differences between groups were mainly in the frontal and parietal regions and the insula, which is consistent with previous studies based on different neuroimaging modes (magnetoencephalography, electroencephalography and fMRI).^31-33^ Specifically, acute mTBI showed increased *Cp* values in left angular gyrus, which surrounds the distal portion of the superior temporal sulcus and is the visual language centre (reading centre). Using diffusion tensor imaging (DTI), Zhou *et al*.^34^ found that the *Cp* of mTBI patients was significantly increased (3–53 days after trauma) and proposed that the increased local *Cp* may reflect a compensatory mechanism based on the upregulation of local networks negatively related to post-concussion symptoms.

In addition to the global level, we also found an effect of acute mTBI on FC at the intermediate module level. Here, mTBI selectively modulates SMN and ON, which may be the main cause of the observed global differences. However, to date, few reports have focused on the modulation between SMN and ON function in mTBI based on CBF, limiting further speculation on this finding. The SMN includes primary and higher somatosensory areas and motor areas and extends to the supplementary motor areas.^35^ The SMN can receive sensory input from the outside world, which is important for somatosensory awareness and the generation of appropriate motor responses.^36,37^ The ON is located in the occipital lobe and is mainly involved in visual processing.^38^ Our results showed the reduced network connectivity between SMN and ON and significant impairment of visuospatial/executive capacity after mTBI, suggesting that intrinsic inactivation and reduced efficiency of SMN and ON may be one of the reasons for the decreased visual spatial/executive capacity after mTBI. However, we did not find a significant association between SMN–ON connectivity of the CBF network and cognitive performance, which may be due to the limited sample size or the severity of cognitive impairment in the mTBI group. On the other hand, perhaps our results can be better interpreted and lead to similar conclusions as the Cassady *et al*.^39^ study, regarding SMN and ON as networks interacting with the outside world. Moreover, the significant changes in FC were unilateral and primarily in the left hemisphere, possibly due to the nature of the effects. Previous studies using a mTBI model of unilateral mouse closed head injury found fractional anisotropy alterations in the contralateral corpus callosum.^40^ Fidan *et al*.^41^ found that the ipsilateral cingulum cortex and contralateral external capsule showed a significant increase in radial diffusivity in the unilateral immature mTBI animals. However, the mechanism of unilateral or ipsilateral FC changes after mTBI remains to be determined.

This study has several limitations. First, this was a cross-sectional study with a limited sample size, which limits the prediction of the relationship between our findings and the development and prognosis of cognitive impairment in patients with mTBI. Second, the trauma localization of mTBI patients and the relationship between trauma localization and local CBF were not analysed in this study. Future studies should control these factors. Third, as a preliminary study, we simply assessed cognitive function using the MoCA. Detailed neurocognitive scales corresponding to different cognitive domains will provide a better understanding of the underlying mechanisms of cognitive impairment after mTBI. Finally, significant correlation between *Eg* and the visuospatial/executive score no longer existed after the FDR correction, although our result still has implications for research in this area.

This preliminary study based on CBF haemodynamic fluctuations revealed that mTBI could affect regional CBF, CBF network connectivity patterns and the topological characteristics of the brain network in the acute stage, and CBF network topology indicators were correlated with cognitive function. Our study highlights the potential use of ASL-based CBF properties in studying the underlying neuropathology of mTBI; thus, they may be an effective neuroimaging index in identifying and evaluating mTBI and post-traumatic cognitive impairment.

## Data Availability

The data that support the findings of this study are available on request from the corresponding author. The data are not publicly available due to privacy or ethical restrictions.
